# Nucleolin Regulates the Expression of Kaposi’s Sarcoma-Associated Herpesvirus’ Latency-Associated Nuclear Antigen through G-Quadruplexes in the mRNA

**DOI:** 10.3390/v15122438

**Published:** 2023-12-15

**Authors:** Andrew R. Zareie, Subhash C. Verma

**Affiliations:** Department of Microbiology and Immunology, University of Nevada, Reno School of Medicine, 1664 N Virginia Street, Reno, NV 89557, USA; azareie@nevada.unr.edu

**Keywords:** G-quadruplex, LANA, KSHV, Nucleolin (NCL)

## Abstract

Kaposi’s sarcoma-associated herpesvirus (KSHV) establishes life-long latent infection and is linked to several human malignancies. Latency-associated nuclear antigen (LANA) is highly expressed during latency, and is responsible for the replication and maintenance of the viral genome. The expression of LANA is regulated at transcriptional/translational levels through multiple mechanisms, including the secondary structures in the mRNA sequence. LANA mRNA has multiple G-quadruplexes (G4s) that are bound by multiple proteins to stabilize/destabilize these secondary structures for regulating LANA. In this manuscript, we demonstrate the role of Nucleolin (NCL) in regulating LANA expression through its interaction with G-quadruplexes of LANA mRNA. This interaction reduced LANA’s protein expression through the sequestration of mRNA into the nucleus, demonstrated by the colocalization of G4-carrying mRNA with NCL. Furthermore, the downregulation of NCL, by way of a short hairpin, showed an increase in LANA translation following an alteration in the levels of LANA mRNA in the cytoplasm. Overall, the data presented in this manuscript showed that G-quadruplexes-mediated translational control could be regulated by NCL, which can be exploited for controlling KSHV latency.

## 1. Introduction

Kaposi’s sarcoma-associated herpesvirus (KSHV) is a DNA virus linked to several human malignancies, including Kaposi’s sarcomas (KS), multicentric Castleman’s disease, and primary effusion lymphoma (PEL) [1,2,3,4]. KSHV establishes a life-long infection with two distinct phases, a prevalent latent phase and a short lytic phase [5,6]. ORF73 encoded latency-associated nuclear antigen (LANA) is predominantly expressed during the latent phase [7,8]. LANA is essential for replication and the maintenance of the viral episome [9]. The virus evades the host’s immune surveillance while maintaining the viral genome by expressing the LANA protein at a level sufficient for maintaining its genome. LANA evades CD8^+^ T-cells by controlling its expression through downregulation of its promoter to suppress LANA expression. CD8^+^ T-cell evasion is also achieved by inhibiting the translocation of LANA peptide to the endoplasmic reticulum and its presentation through MHC-I [10,11,12]. We previously showed that LANA also inhibits CD4^+^ T-cell response by downregulating the MHC-II gene by blocking CIITA transcription through its interaction with the regulatory factor X (RFX) complex [13].

**Figure 1:**
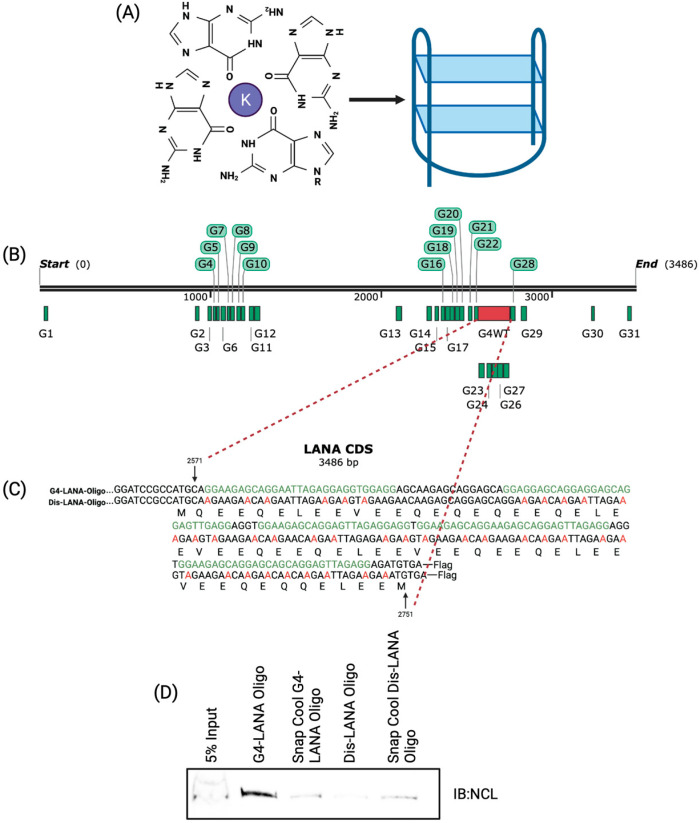
G-quadruplexes in LANA mRNA. (**A**) Four guanines stabilized by non-canonical hydrogen bonding and a potassium ion form a G-tetrad. On the right, three G-tetrads stacked upon one another, further stabilized by π−π bonding, forming a G-quadruplex. (**B**) LANA mRNA showing the locations of all the potential non-overlapping G-quadruplexes identified through QGRS Mapper. G4 sites are represented by “G” and then the number indicating the order of the G4s in LANA mRNA. The red region indicates the G4WT region encompassing G4 sites, G23–G27. (**C**) Sequences of G4WT and G4Dis constructs. In the G4WT sequence, potential G4 sites are in green. In the G4Dis sequence, critical Gs have been changed to As to eliminate the G4 formation while maintaining the amino acid sequence. (**D**) Immunoblot blot (IB) showing Nucleolin from affinity pulldown assay with wild-type (WT) LANA oligo. Biotinylated WT-LANA or Dis-LANA oligonucleotides were heated to 95 °C and allowed to slowly cool by 1 °C per minute until room temperature was reached to enable the formation of G4 or snap cooled on ice post-heating to prevent G4 formation. Oligos were incubated with cellular lysate from HEK293L cells for 24 h, then incubated with streptavidin beads to pulldown the RNA-bound proteins. Samples were resolved on an SDS-PAGE and immunoblotted with NCL antibody.

This study focuses on G-quadruplexes (G4s), non-canonical secondary structures in guanine-rich DNA and RNA sequences. G4s are formed when two or more consecutive guanine blocks are separated by a single-stranded loop region [14]. Four consecutive runs of guanine blocks result in G-tetrads held together by Hoogsteen hydrogen bonding between the guanines (Figure 1A). These structures fold into two or more stacked planar G-tetrads, and stability is increased by way of π-π stacking between the quartets, and further stabilized by a cation, such as potassium [15]. The stability of G4s is increased with more quartets stacked and lower numbers of nucleotides comprising the loop regions. G4s are energetically favorable, with a negative ΔG, and form spontaneously [14,16,17,18,19]. The G4s serve as regulatory elements in transcription, translation, RNA maturation, and regulation of non-coding RNA [20]. Potential G4 sequences are more likely to fold into a secondary structure in RNA due to the lack of a complementary strand compared to DNA. Notably, the folding of potential G4-forming sequences results in more stable RNA molecules [15]. G4 sequences are present in functional domains of mRNA, including 5′-UTR, ORFs, and 3′-UTR [21,22,23]. G4s can affect the genome’s coding capacity through alternative polyadenylation, alternative splicing, and induced frameshifts [20]. G4s can be regulated by either stabilizing or unwinding the secondary structure [21]. Recently, G4s gained interest as potential therapeutic targets against pathogenic viruses. For instance, the Hepatitis B virus contains a G4 that positively regulates its transcription, and disruption of this G4 reduces virion production [24]. Human immunodeficiency virus (HIV-1) has a G4 that reduces the transcriptional activation of HIV [25]. Stabilization of a G4 in Herpes Simplex Virus 1 resulted in reduced virion production [26]. The G4 in Epstein–Barr virus (EBV) nuclear antigen (EBNA1) can regulate translation and antigen presentation [27].

Previously, our lab reported the formation of a G4 in the terminal repeat of KSHV and its impact on latent viral replication [28]. We also showed that the mRNA of LANA has multiple G4 sites (Figure 1B), and the disruption of these sites led to increased LANA translation and antigen presentation [29]. Notably, the expression of EBV’s latent antigen, EBNA1, a homolog of LANA, is maintained at optimal levels for viral episome replication/maintenance [27,30,31]. Interestingly, EBNA1 has a glycine-alanine repeat (GAr) region, which forms G-quadruplex structures to regulate its expression through interaction with Nucleolin (NCL) [27]. NCL is a multifunction DNA/RNA-binding protein that is conserved among eukaryotes. NCL binds to the mRNAs of many cancer-related genes to regulate their translation [32]. For example, NCL binds to G-quadruplexes formed in the LTR promoter of HIV, resulting in the downregulation of viral transcription [33]. Additionally, NCL binds and stabilizes a G-quadruplex within the c-MYC promoter, affecting HIV transcription [34].

Several open-source programs based on G-quadruplex prediction algorithms are available for detecting putative G4-forming sequences (PQSs). Noteworthy examples include G4Hunter, Quadparser, and QGRSMapper. G4Hunter and Quadparser identify a relatively smaller number of G4s and require programming skills for their usage. On the other hand, QGRSMapper can identify a greater number of potential G4s and operates as a standalone web-based program [35]. The potential G-quadruplex sites can be determined in silico using QGRS Mapper, which led to the identification of multiple G-quadruplex forming sites in LANA [20,29,35,36,37,38,39,40,41,42,43,44,45]. Furthermore, the role of these G-quadruplexes in regulating LANA expression through the helicase activity of hnRNP A1 in unwinding these G4s was demonstrated earlier [29]. In this paper, we set out to determine whether NCL plays a role in regulating the expression of LANA, and the data presented here convincingly show that NCL controls LANA expression through G-quadruplexes.

## 2. Materials and Methods

### 2.1. Cells

Human kidney (HEK 293L) cells maintained using Dulbecco’s Modified Eagle’s Medium (DMEM) supplemented with 8% BGS (Hyclone Laboratories, Logan, UT, USA), 2 mM L-glutamine (GE Healthcare Life Sciences, Marlborough, MA, USA), 25 U/mL penicillin (GE Healthcare Life Sciences, Marlborough, MA, USA), and 25 µg/mL streptomycin (GE Healthcare, South Logan, UT, USA). HEK293KbC2 cells were a generous gift from Johnathan Yewdell (NIH) and maintained in DMEM with 8%BGS, 2 mM L-glutamine, 25 U/mL, 25 U/mL penicillin, and 25 µg/mL streptomycin. T cell hybridoma line (B3Z), a kind gift from Nilabh Shastri (John Hopkins University, Baltimore, MD, USA) and Charles L. Stentman (Dartmouth University, Hanover, NH, USA), were grown in RPMI with 10% FBS, 2 mM glutamine, 1mM pyruvate, 25 U/mL penicillin, and 25 µg/mL streptomycin. Human epithelial (HELA) cells were grown in DMEM supplemented with 10% FBS, 2 mM L-glutamine, 25 U/mL penicillin, and 25 µg/mL streptomycin. pA3F-LANA, LANA-luciferase, pA3F-G4WT, pA3F-G4Dis, pA3F-LANA-OVA, pA3F-G4WT-OVA, pA3F-G4Dis-OVA have been described previously in (Verma 2013, Purushothaman 2012, Dabral 2021) [29,46,47].

Cell transfections were carried out either by polyethylamine (PEI) or Lipofectamine 3000. For a 6-well plate containing 1 million cells/well, 3 µg of the desired plasmid was mixed with 180 of 150 mM NaCl (Fisher Scientific, Waltham, MA, USA), and 15 µL (1 mg/mL) PEI was incubated for 15 min before adding the solution to the well. After 4 h of incubation, media was aspirated off, and new media was added to the cells. At 24 h post-transfection, the cells were used for the indicated experiments. Scaling of components was done according to well size: 10 cm TC dish (900 µL 150 mM NaCl with 75 µL 1 mg/mL PEI), 12-well plate (90 µL 150 mM NaCl with 7.5 µL 1 mg/mL PEI), 24-well plate (45 µL 150 mM NaCl with 3.75 µL 1mg/mL PEI). Transfection using Lipofectamine 3000 (Thermofisher, Waltham, MA, USA) was done as per the manufacturer’s protocol.

### 2.2. Dual Luciferase Assay

The Dual-Luciferase Reporter Assay System (Promega Inc., Madison, WI, USA) was used for the luciferase assays. Cells were lysed with 100 µL of 1× passive lysis buffer for 15 min, with gentle rocking. A total of 20 µL of the lysate was collected and transferred into a 96-well plate. A total of 20 µL of LAR reagent was added to the lysate to detect Firefly luciferase activity. Renilla luciferase activity was measured by adding 20 µL of Stop&Glo reagent. The data were analyzed and presented as the ratio of Firefly/Renilla.

### 2.3. Fluorescent In Situ Hybridization (FISH)

Fluorescent in situ hybridization was performed as described previously (Kochan et al. 2015) [48]. Briefly, the cell monolayers were fixed directly to the coverslip using 4% paraformaldehyde (Sigma-Aldrich, St. Louis, MO, USA) for 15min at room temp (RT). Following 3× wash with PBS (Life Technologies, Hewlett, NY, USA) with 5 min of gentle rocking, cells were permeabilized for 10min while shaking with 1% BSA (Sigma, MO), 0.3% Triton X-100 (Fisher Scientific, Waltham, MA, USA), 2 mM Vanadyl ribonucleoside complexes (Sigma, MO), 1× PBS, and DEPC-treated water (Sigma-Aldrich, St. Louis, MO, USA). These cells were washed 3× with PBS for 5 min while rocking gently. A total of 200 nM of biotinylated OVA-oligonucleotide (5′-UCCAUCAUCAAUUUCGAGAAACUC-biotin-3′), WT LANA RNA oligonucleotide (WT-LANA-Oligo) (5′-UGGAAGAGCAGGAAGA GCAGGAGUUAGAGGA-biotin-3′) or Dis-LANA RNA oligonucleotide (Dis-LANA-Oligo) (5′-UAACCGAUGAUAUGAGUC AGAUAUAUAAGCA-biotin-3′) (IDT, IA) was added in 2× SSC (diluted from 20× SSC: NaCl 3 M and trisodium citrate dihydrate 300 nM), 10% formamide (Fisher Scientific, Waltham, MA, USA), 10% dextran sulfate (Sigma-Aldrich, St. Louis, MO, USA) DEPC-treated water for hybridization. Following an overnight Incubation at 37 °C, cells were washed 3× with gentle rocking in PBS for 5 min. A total of 5 µL of NCL antibody Cat#PA3-16875 (Invitrogen, Carlsbad, CA, USA) was added into 1 mL of permeabilization solution for immune localization by using 200 µL of the mixture to each coverslip. Following overnight incubation at 37 °C, coverslips were washed 3× with PBS for 5 min/each with gentle rocking. Alexa Fluor streptavidin-594 (Invitrogen, Carlsbad, CA, USA) and Alexa Fluor 488 (Invitrogen, Waltham, MA, USA) added to the permeabilization buffer were used for the localization of the biotin-labeled probe and NCL, respectively. Streptavidin Alexafluor-594 and Alexa Fluor 488 mix were added onto the coverslips and incubated for 1 h at 37 °C, followed by 3× PBS washes for 5min/each with gentle rocking. The nuclei were stained with DAPI (Invitrogen, Carlsbad, CA, USA) or ToPro3 (Thermofisher, Waltham, MA, USA) for 1 min at RT and washed 3× with PBS for 5 min before mounting with antifade (Invitrogen, Carlsbad, CA, USA) onto a slide. These slides were dried overnight before imaging on a Zeiss Imager M2 with Zen 2012 software. BCBL-1 cells are subjected to the same protocol, except that they are fixed to the coverslips using 10% formaldehyde for 10 min, following a 30 min incubation in PBS on the coverslips.

### 2.4. Antigen Presentation Assay

HEK293Kbc2 cells were transfected with LANA-OVA, G4WT-OVA, or G4Dis-OVA plasmids. At 24 h post transfection, the cells were incubated with B3Z cells for 18 h at a ratio of 1:2, 1:1, and 1:0.5. Cells were incubated with buffer containing 0.124% NP-40, 9 mM MgCl_2_, and 5 mM ONPG for 4 h at 37 °C with 5% CO_2_. Absorbance was measured at 450 nM GloMax Explorer microplate reader (Promega, Madison, WI, USA). For the MTT Assay, the same ratio of cells as the antigen presentation is used and pipetted into a 96-well plate. After incubating for 18 h, cells were incubated with 150 µL of MTT (Invitrogen, Carlsbad, CA, USA) for four hours. The absorbance was measured at 560 nM using a Hidex Chameleon (Hidex, Turku, Finland) microplate reader at 570 nM.

### 2.5. RNA Cross-Linking Immunoprecipitation Assay (RNA-CLIP)

RNA-CLIP was modified as described previously [49]. Briefly, 5 million cells were transfected in 10 cm TC dish with LANA-OVA, G4WT-OVA, or G4Dis-OVA plasmids. At 24 h post-transfection, the cells were fixed with 4% formaldehyde and quenched with the addition of 125 mM glycine. Cells were then washed and pelleted. Following resuspension in DNA/RNA Shield (Zymo Research, Irvine, CA, USA). The nuclei were isolated by sonicating cells at 30 W, 20 s on, 30 s off, for a total runtime of 7 min and 30 s. Chromatin was sheared between 200–400 bp using QSonica CL-334 Sonicator (Fisher Scientific, Waltham, MA, USA), following which the cell debris was pelleted. The chromatin was incubated with specific antibodies overnight, followed by a 1 h protein A/G magnetic Sepharose bead incubation to capture the protein-RNA complex. The beads were washed once with low-salt buffer (0.1% SDS, 1% Triton X-100, 2 mM EDTA, 20 mM Tris [pH 8.0], 150 mM NaCl) and once with high-salt buffer (0.1% SDS, 1% Triton X-100, 2 mM EDTA, 20 mM Tris [pH 8.0], 500 mM NaCl). Beads were washed with 1 mL TE, and a 10% aliquot was set aside for Western blotting. Beads were resuspended in 150 µL of elution buffer (1% SDS, 100 mM NaHCO_3_), with gentle vortexing for 15 min to elute the complex. Elutions were reverse cross-linked overnight at 65 °C using 0.3 M NaCl. The following day, RNA was extracted using Direct-zol RNA Miniprep Plus (Zymo Research, Irvine, CA, USA) per the manufacturer’s instructions.

### 2.6. In Vitro RNA Pulldown Assay

WT LANA RNA oligonucleotide (5′-UGGAAGAGCAGGAAGA GCAGGAGUUAGAGGA-biotin-3′) (IDT, IA) and Dis-LANA-Oligo RNA oligonucleotide (5′-UAACCGAUGAUAUGAGUC AGAUAUAUAAGCA-biotin-3′). Twenty million HEK293L cells were lysed in 1% NP-40 lysis buffer (50 mM Tris-HCl pH7.5, 150 mM NaCl, 1% NP-40, 1 mM EDTA pH 8.0) with protease inhibitors and RNaseOut. Prior to incubation, oligos were heated to 95 °C for 5 min to allow G4 formation, then cooled at 1 °C per minute until 25 °C. A second set of oligos were snap-cooled after the 95 °C incubation to prevent G4 formation. Cells were sonicated and centrifuged to remove cellular debris. RNA oligos were incubated with the cellular lysate overnight while rotating at 4 °C. Pierce streptavidin-agarose beads (ThermoFisher Scientific, Waltham, MA, USA) were added to the samples for 2 h to pulldown proteins bound to the RNA oligos. Beads were washed thrice with 1%NP-40 lysis buffer, loaded onto a 9% SDS gel, and then transferred onto a nitrocellulose membrane. The membrane was incubated with anti-NCL antibodies, followed by the secondary antibodies labeled with IR dye. The membrane was scanned using the LI-COR Odyssey Imaging System.

### 2.7. Fractionation and qRT-PCR

HEK293L cells are transfected with shNCL (Horizon Discovery, Cambridge, UK) plasmid and incubated with and without 1 µg/mL final concentration doxycycline for 24 h prior to transfection. Cells are then transfected with G4WT-OVA or G4Dis-OVA and allowed to incubate overnight. At 24 h post-transfection, cells were washed with PBS then lysed in 300 µL Nuclear Extraction Buffer (NEB: 20 mM HEPES pH 7.2, 50 mM NaCl, 3 mM MgCl_2_, 300 mM sucrose, and 0.5% NP-40) by incubating for 15min on ice after resuspending the cells. The nuclei were pelleted at 800× *g*, 10 min, 4 °C, followed by collecting the cytoplasmic fraction in a separate tube. The nuclei were washed twice with PBS and resuspended in 300 µL NEB to sonicate for 10 s on and 10 s off twice. The debris was removed by centrifuging at 800× *g*, 10 min, 4 °C. RNA was extracted using Trizol-LS (Life Technologies, Carlsbad, CA, USA) and Direct-zol RNA Miniprep Plus (Zymo Research, Irvine, CA, USA), as per the manufacturer’s instructions. cDNA synthesis was performed using a High-Capacity RNA-to-cDNA Kit (Applied Biosystems, Vilnius, Lithuania) and quantified using specific primers with SsoAdvanced Universal SYBR Green Supermix (Biorad, Hercules, CA, USA) on a Quant Studio 5 (Thermo Scientific, Waltham, MA, USA).

### 2.8. Statistical Analysis

*p* values were calculated by a two-tailed *t*-test using GraphPad Inc. (Prism 8) software for statistical significance. In the figures, asterisks represent *p* values as follows: *, *p* value < 0.05; **, *p* value < 0.01; ***, *p* value < 0.001; and ****, *p* value < 0.0001, and “ns” is no statistical significance. All experiments were performed at least three times with three biological replicates where applicable.

### 2.9. Figure Generation

Figures were generated using Prism 8, ImageJ, Adobe Illustrator, Adobe Photoshop, SnapGene software (www.snapgene.com, accessed on 17 January 2022), and Biorender.com (accessed on 27 September 2023).

## 3. Results

### 3.1. Nucleolin Binds to the G-Quadruplex of LANA mRNA

G-quadruplexes are essential regulatory elements that can modulate a variety of biological processes. The expression of EBV’s EBNA1, a functional homolog of LANA, has been shown to be regulated by G4s in its mRNA [50]. In silico mapping of LANA mRNA through QGRS Mapper identified multiple G-quadruplex in the QE domain of LANA (Table S1), which can be bound by a cellular RNA helicase, hnRNP A1, to modulate the expression of LANA protein [29]. Crucially, EBNA1 expression was shown to be regulated by NCL, a multifunctional DNA/RNA binding protein that stabilizes the G4 structure through direct binding [27]. Here, we sought to determine whether NCL plays a role in regulating the expression of LANA through the G-quadruplexes in its mRNA. To this end, we first tested the interaction of NCL with LANA mRNA by performing an RNA-cross-linking immunoprecipitation assay (RNA-CLIP). We used RNA oligonucleotides of a portion of the LANA mRNA with G4 sites as wild-type (WT) oligonucleotide along with an oligonucleotide of the same length with disrupted G4 (Dis) sites as a control oligonucleotide (Figure 1C). The WT LANA oligonucleotide forms a stable G4, but not the control (Dis) oligonucleotide [29]. Cell lysate from the HEK293L was incubated with biotinylated WT or Dis-LANA-Oligo oligonucleotides for 24 h, followed by precipitation of the oligonucleotide-bound proteins with Streptavidin Sepharose beads. A set of oligonucleotides were snap-cooled to prevent the formation of G4s and used in the pulldown assay as another control. Samples were subjected to immunoblot to detect any NCL bound to the G-quadruplexes (Figure 1D). Our data showed that NCL binds to the WT-LANA-Oligo more efficiently than the Dis or snap-cooled oligos demonstrated by an efficient pulldown of NCL with G4 forming oligo. This suggested that NCL can interact with the G4s of LANA mRNA. The fainter band of NCL in the Dis-LANA-Oligo lane could be because NCL is a DNA/RNA binding protein, and the Dis-LANA-Oligo oligonucleotide may have some necessary characteristics for its binding to NCL. However, it is essential to emphasize that the WT-LANA-Oligo consistently showed higher affinity to dNCL than the Dis-LANA-Oligo.

**Figure 2:**
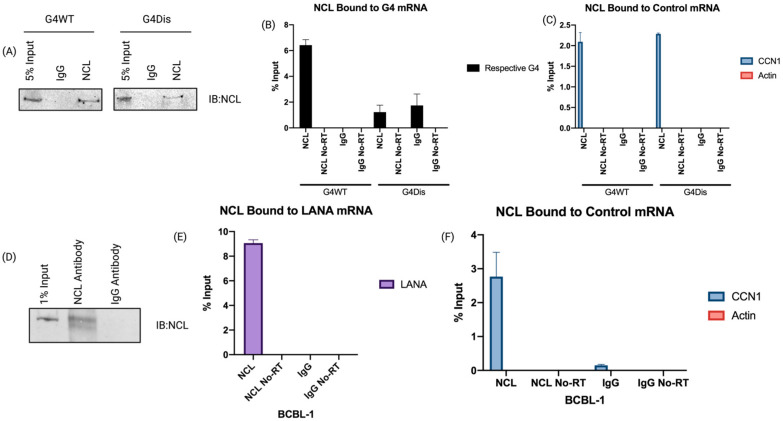
Nucleolin binds to the G-quadruplexes in LANA mRNA. RNA-CLIP assay confirming the direct interaction between LANA mRNA and NCL. HEK293L cells were transfected with G4WT-ova and G4Dis-ova plasmids and harvested 24 h post-transfection. Cells were cross-linked, lysed, and incubated with anti-NCL antibodies and magnetic protein A/G beads. RNA bound to the beads was purified and quantified in two steps using a High Capacity RNA-to-cDNA Kit followed by SsoAdvanced Universal SYBR Green Supermix with G4WT and G4Dis clone-specific primers. (**A**) Immunoblot showing a specific pulldown of NCL with anti-NCL antibody compared to the control antibody, IgG. (**B**) qRT-PCR data showing relative binding of G4-WT or G4-Dis RNA in the RNA-CLIP assay. (**C**) qRT-PCR data showing relative binding of CCN1 to NCL, used as a positive control and Actin as a negative control. (**D**) RNA-CLIP from KSHV-infected cells, BCBL-1. NCL was specifically immunoprecipitated with anti-NCL antibodies but not control IgG, as expected. (**E**) Relative binding of LANA mRNA bound to NCL compared to the control IgG. (**F**) qRT-PCR data of relative CCN1 and Actin mRNA bound to NCL in BCBL-1 cells. All qPCR rxns were ran with “no reverse transcriptase” (No RT) as a control.

Furthermore, we wanted to see if NCL binds to LANA mRNA in vivo, which we investigated by using our previously designed constructs with a 250-nucleotide long QE region of LANA encompassing the G-quadruplex forming region (G4WT) (Figure 1B,C) [29]. We used a codon-optimized construct with a disrupted G4 (G4Dis) site as a control while maintaining the amino acid sequence identical to the G4WT [29]. The constructs with the ovalbumin epitope (SIINFEKL) downstream of the full-length LANA (FL-LANA-ova), G4WT (G4WT-ova), and G4Dis (G4Dis-ova) were transfected in HEK293L cells, and 24 h post-transfection, the NCL-bound complexes were immunoprecipitated followed by cross-linking, using an anti-NCL and a control, IgG antibodies. RNA-protein complex was sheared to approximately 300 bp, followed by immunoprecipitation with magnetic protein A/G beads. A fraction (10%) of the immunoprecipitated complex was subjected to SDS-PAGE/immunoblot to confirm the immunoprecipitation of NCL protein (Figure 2A). Results indicated that the NCL antibody efficiently pulldown comparable levels of NCL protein from cells transfected with G4WT and G4Dis constructs, as expected. Control, IgG antibody did not precipitate any detectable levels of NCL from either set, confirming specific precipitation of NCL with anti-NCL antibodies. To determine the levels of NCL-bound RNA, the immunoprecipitated complexes were subjected to RNA extraction and cDNA synthesis to quantify G4WT or G4Dis mRNA using gene-specific primers. The relative levels of NCL-bound G4WT mRNA were calculated in reference with respective inputs (Figure 2B). Our data showed a significant binding of G4WT mRNA to NCL, presented as percent input as compared to the G4Dis mRNA. G4Dis mRNA and Icontrol IgG showed significantly lower levels of binding, confirming the specific association of NCL with G4 containing mRNA. RNA without Reverse-Transcriptase (No RT) did not show specific amplification confirming the binding of NCL with the mRNA. To substantiate the interaction between NCL and the G4 sequences, we evaluated the levels of Cyclin I (CCNI) mRNA binding to NCL in our assay, given its already documented association with NCL (Figure 2C) [32]. We found specific binding of CCNI with NCL precipitated from both G4WT and G4Dis samples, confirming the specificity of NCL’s binding to the G4s. Additionally, we checked the binding of a control mRNA, actin, which has not been shown to interact with NCL, showed no binding (Figure 2C). We further sought to determine whether this interaction occurred in KSHV-infected PEL (primary effusion lymphoma) cells; which we did by utilizing the BCBL-1 cell line. Latently infected BCBL-1 cells were cross-linked and subjected to RNA-CLIP as above. Immunoprecipitation of NCL was confirmed by anti-NCL immunoblot (Figure 2D), followed by the extraction of RNA bound to NCL for quantification of LANA mRNA using specific primers. Relative quantitation compared to the input showed a significant binding of LANA mRNA with NCL as compared to the control antibody, IgG (Figure 2E). RNA with No-RT did not show any amplification, confirming the specificity. Importantly, we found comparable levels of CCN1 mRNA binding to NCL in KSHV-infected as well as transfected (G4WT and G4Dis) cells. Similarly, comparable levels of NCL binding to G4WT mRNA and LANA mRNA binding were detected in both systems. Similar to the transfection system, actin mRNA did not show any binding in BCBL-1 cells (Figure 2F). These results confirmed that NCL specifically binds to the G-quadruplex region present in LANA mRNA.

We wanted to demonstrate the importance of G4 sites of full-length LANA mRNA by ablating all the G4 sites, without affecting the amino acid sequence, but failed to generate such a construct because of the presence of highly repetitive GC-rich region in LANA, which led to the re-introduction of G4 sites following codon optimization after scrambling G4 sequences.

### 3.2. Increasing Expression of NCL Inhibited LANA Translation

**Figure 3:**
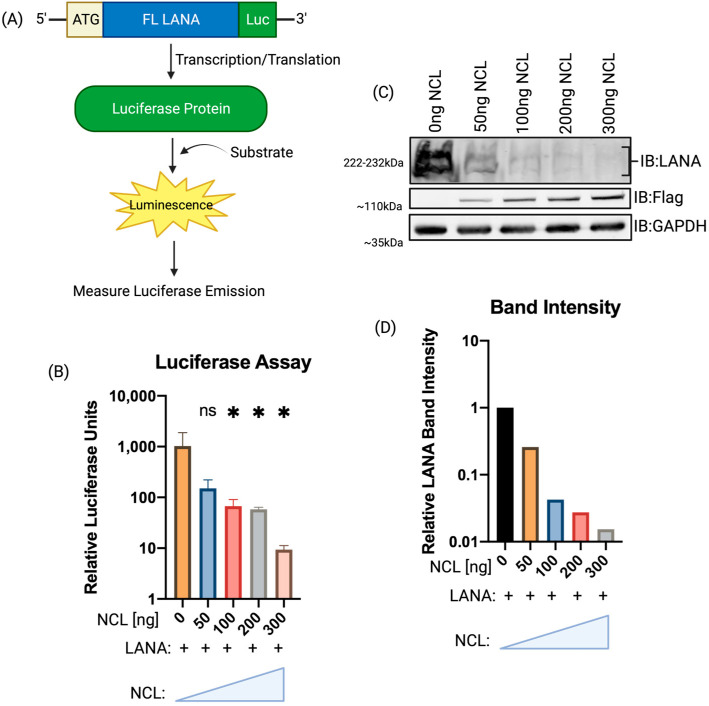
Nucleolin regulates LANA translation. (**A**) A flowchart of luciferase assay along with LANA construct. The coding sequence of LANA was subcloned into a pGL3 vector upstream of the luciferase gene in-frame to produce a LANA-luciferase fusion protein. (**B**) HEK293L cells were transfected with a constant amount of LANA-luciferase and increasing amounts of NCL-expressing plasmid. At 24 h post-transfection, determination of relative luciferase units showed a decrease in luciferase levels with increasing amounts of NCL. (**C**) Immunoblot showing an increasing level of NCL (Flag tagged) with constant levels of LANA cotransfected. LANA presents as a “ladder,” detected as two bands. GAPDH immunoblot was used as a control. (**D**) Graphical depiction of LANA band intensities. Levels of LANA were normalized to 0 ng NCL sample. The asterisk (*) represents the significance level of the relative band intensity below 0 ng NCL sample. “ns” represents no statistical significance. The “+” symbol indicates samples transfected with LANA. The triangle indicates increasing concentration of NCL transfected from left to right.

NCL can bind to G4 to reduce the availability of mRNA for translation; therefore, we sought to determine whether NCL overexpression altered the expression of LANA protein. Using a full-length LANA construct with Luciferase fused downstream in-frame (Figure 3A), HEK293L cells were cotransfected with increasing amounts of NCL. At 24 h post-transfection, cells were lysed for the quantification of luminescence, which showed a gradual decrease in luminescence with increasing NCL levels (Figure 3B). Reduction in luminescence, an indirect measure of LANA expressions, was further validated by detecting the LANA protein levels through immunoblotting (Figure 3C). Band intensities of LANA with increasing levels of NCL were quantified via pixel density of these bands using ImageJ (Figure 3D). Band intensities of LANA, normalized to respective GAPDH band intensities, showed a dose-dependent reduction in LANA expression, supporting our hypothesis that NCL can reduce LANA translation by restricting the translocation of LANA mRNA.

### 3.3. LANA mRNA Colocalizes with NCL

**Figure 4:**
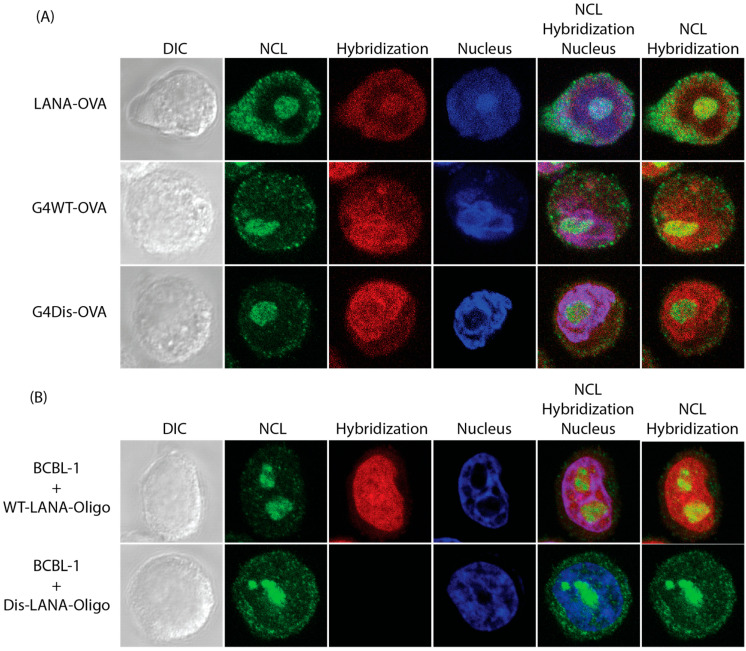
RNA Fluorescent in situ hybridization. (**A**) HeLa cells were transfected with a plasmid expressing G4WT or FL-LANA. At 24 h post-transfection, cells were fixed, permeabilized, and incubated with biotinylated ovalbumin oligonucleotide sequence. Cells were further incubated with anti-NCL antibodies to localize NCL using goat anti-rabbit Alexa Four 488 antibody (green). Biotinlated hybridized oligos were detected using Streptavidin Alexa Fluor 594 (red). Nuclei were stained with TOPRO-3 (blue). (**B**) BCBL-1 cells hybridized with G4-specific oligos (WT-LANA-Oligo) or the control (Dis-LANA-Oligo). Images were taken with a Zeiss confocal microscope at 100× magnification. Exposure settings were constant between WT-LANA-Oligo and Dis-LANA-Oligo.

We further wanted to determine whether LANA expression decreased due to the binding of LANA mRNA with NCL. To address this, we performed RNA fluorescent in situ hybridization (FISH) [48] of mRNA with G4 sites using constructs with the ovalbumin epitope (SIINFEKL) downstream of the full-length LANA (FL-LANA-ova) and the previously discussed G4WT-ova and G4Dis-ova. These plasmids were transfected in a monolayer of HeLa cells cultured onto coverslips. Cells were fixed with 4% formaldehyde, permeabilized, and incubated with a blocking buffer 24 h post-transfection. Following, cells were hybridized with biotinylated ovalbumin oligonucleotide sequence overnight. Cells were subsequently incubated with anti-NCL antibodies overnight, followed by detection with Alexa Fluor 488. Biotin-labeled probes were detected by Streptavidin Alexa Fluor 594 and the nuclei with TOPRO-3. We also used latently infected KSHV-positive BCBL-1 cells alongside these samples to determine the colocalization of LANA mRNA with NCL. Our results showed that NCL colocalizes with a subset of LANA mRNA as well as the full-length LANA mRNA (Figure 4A). Hybridization signals showing LANA mRNA were seen throughout the cells with defined puncta within the nucleus. The merge panels of NCL and the hybridization signals for mRNA showed a colocalization demonstrated by the yellow signals (Figure 4, lane title “NCL” and “Hybridization”). We also calculated the colocalization coefficients using FIJI software in conjunction with the “Just Another Colocalization Plugin” (JACoP). These coefficients ranged from 0 for no colocalization to 1 for complete colocalization [51,52]. Our results showed FL-LANA-ova to have a score of 0.606, G4WT-ova with 0.638, and G4Dis-ova 0.312. This suggested colocalization of LANA mRNA with NCL in the nucleus. The BCBL-1 cells were subjected to the FISH assay but were hybridized with either WT-LANA or Dis-LANA oligonucleotides cells showed a comparable degree of colocalization of 0.780 with the WT-LANA-Oligo and 0.07 for the Dis- Oligo (Figure 4B). The colocalization coefficients were averaged from ten images. In the case of FL-LANA and G4WT transfected cells, and BCBL-1 cells, there is a higher colocalization index compared to G4Dis, suggesting the presence LANA mRNA and NCL in the same cellular compartments.

### 3.4. Interaction of LANA mRNA with NCL Decreases Antigen Presentation

**Figure 5:**
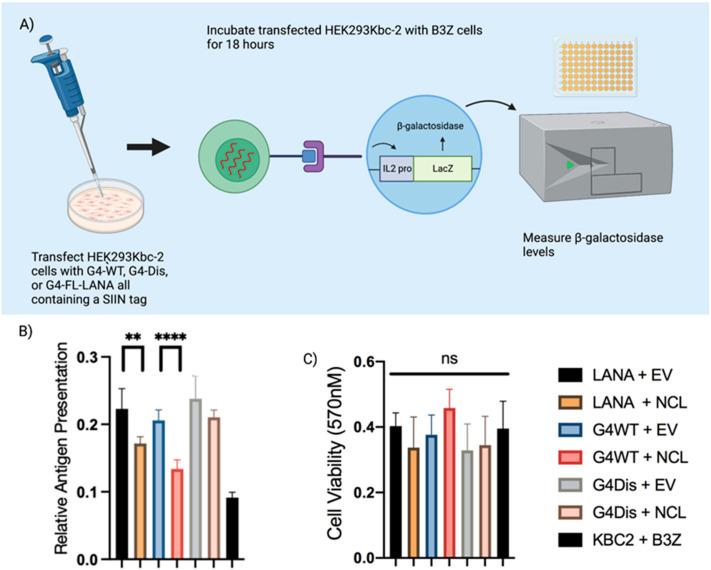
Nucleolin stabilizing G-quadruplex in LANA mRNA decreases antigen presentation. (**A**) A schematic of the antigen presentation assay. Transfected antigen-presenting HEK293Kbc2 cells with a construct encoding sequence-specific ovalbumin epitope, SIINFEKL, with effector T cell hybridoma B3Z cells capable of recognizing the ovalbumin epitope. This interaction results in β-galactosidase activity, which is measured to determine the T cell activation quantitatively. (**B**) T-cell activation from cells transiently expressing FL-LANA, G4WT, and G4Dis. HEK293Kbc2 cells were transfected with pA3F-FL-LANA-ova, pA3F-G4WT-ova, and pA3F-G4Dis-ova (all ovalbumin tagged). At 24 h post-transfection, cells were incubated with B3Z cells for 18 h, then ONPG was added to the cells, followed by measuring β-galactosidase activity at 450 nm. (**C**) MTT assay to measure the metabolic activity of these cells. Asterisks represent *p* values as follows: **, *p* value < 0.01 and ****, *p* value < 0.0001, and “ns” is no statistical significance.

LANA can evade the host’s immune surveillance system by inhibiting various components of the MHC class I & II antigen presentation pathways. Previously, we showed that LANA regulates its expression by recruiting hnRNP A1 to the G4 sites for controlled expression of LANA and its presentation on the cell surface. Since NCL could bind to the G4s in LANA, we aimed to determine the presentation of LANA peptide through antigen presentation assay in the presence of NCL. Previously described ovalbumin constructs were cotransfected with either pA3F-NCL or pA3F-vector into HEK293Kbc2 antigen-presenting cells. At 24 h post-transfection, cells were incubated with T-cell receptor cells (B3Z) for 18 h and then incubated with an ONPG substrate for 4 h. The β-galactosidase activity was then quantified by measuring the optical density (OD) at 450 nm (Figure 5A). Our data showed that expression of NCL decreased antigen presentation of FL-LANA-ova and G4WT-ova transfected cells compared to cells with endogenous levels of NCL (Figure 5B). Cell transfected with the G4Dis-ova did not show any alteration in the levels of antigen presentation. A metabolic assay of the cells transfected with the above construct was performed to ensure that there were not any adverse effects of these proteins in the cell’s metabolism, which may have affected the assay. Our assay (MTT) data showed no statistically significant differences among these samples (Figure 5C). This confirmed that NCL plays a regulatory role in antigen presentation for KSHV’s LANA.

### 3.5. Downregulation of Nucleolin Resulted in an Altered LANA mRNA Localization and Increased LANA Expression

**Figure 6:**
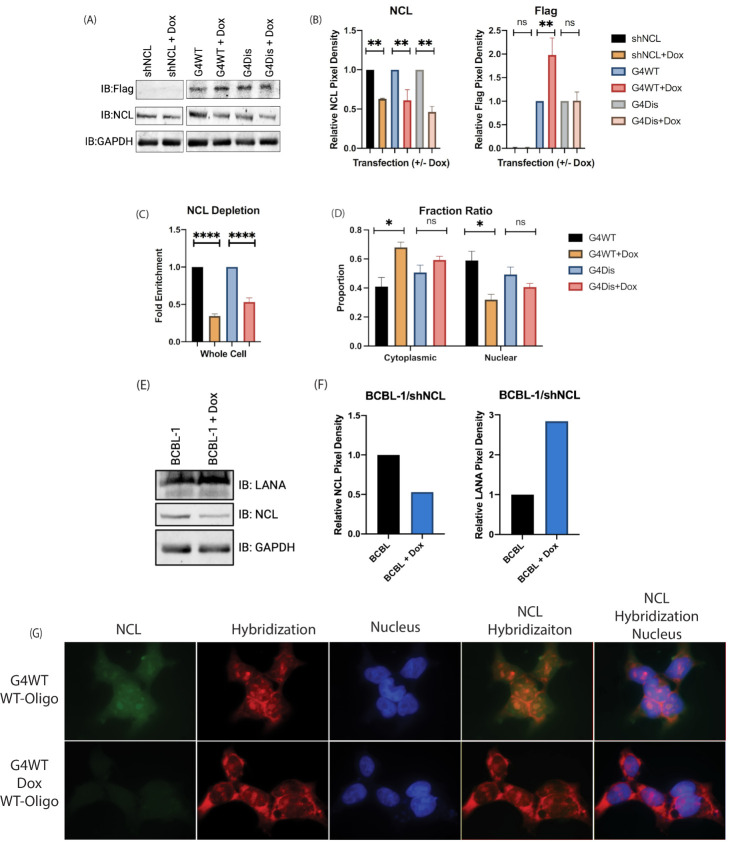
Nucleolin depletion enhanced the expression of LANA mRNA containing G-quadruplexes. (**A**) Immunoblot showing the levels of NCL (IB: NCL) and LANA regions (IB: Flag) in doxycycline and untreated cells transfected with G4 WT or G4 Dis plasmids. Untransfected cells were used as control. GAPDH was used as the loading control. (**B**) Graphical representation of the pixel density of the Western blot in panel “(**A**)”. (**C**) NCL mRNA levels in shNCL transfected cells treated with and without doxycycline (Dox). Samples normalized to GAPDH. (**D**) The ratio of G4WT/G4Dis mRNA copies in the cytoplasmic and nuclear fraction in cells with depleted NCL. The ratio of G4WT mRNA in the cytoplasm of NCL-depleted (Dox) cells was increased compared to those without Dox. Samples were normalized to GAPDH. A proportionate reduction in G4WT mRNA in the nuclear fraction was seen in NCL-depleted cells. The distribution of G4Dis mRNA in the cytoplasmic and nuclear fractions was largely unaffected. (**E**) Immunoblot showing LANA(IB: LANA) levels in BCBL-1 transfected with shNCL and treated with and without doxycycline. (**F**) The left graph shows the relative pixel density of NCL normalized to their respective GAPDHs and then to untreated BCBL-1 cells. The graph on the right shows the relative pixel density of NCl normalized to their respective GAPDHs and then to the untreated BCBL-1 cells. (**G**) Localization of NCL and G4WT mRNA through FISH. shNCL-HEK293L cells cotransfected with shNCL and G4WT plasmid; then treated with and without Dox were fixed, permeabilized, and incubated with biotinylated WT-LANA oligonucleotides. These cells were further incubated with anti-NCL antibodies followed by Alexa Fluor secondary antibodies 488 nm (goat anti-rabbit) and Alexa Fluor 594 (streptavidin). Images were taken with a fluorescent microscope at 100× magnification. The green color represents the localization of NCL, red for oligonucleotide hybridizing with LANA plasmids, and blue for the nucleus. Asterisks represent *p* values as follows: *, *p* value < 0.05; **, *p* value < 0.01; and ****, *p* value < 0.0001, and “ns” is no statistical significance.

RNA interference (RNAi) is a post-transcriptional mechanism for regulating gene expression and has been extensively used for altering protein levels through the introduction of short hairpin RNA (shRNA) [53,54,55]. shRNA are RNA molecules with a hairpin, which are cleaved to generate siRNA for downregulating protein expression [56]. Since increasing NCL resulted in a reduced LANA translation, we sought to determine the effects of NCl downregulation on LANA expression through transfection of HEK293L cells with short hairpin NCL (shNCL) plasmid. Treatment with 1 µg/mL of doxycycline (Dox) to induce shRNA expression, we saw an increase in G4WT protein levels (Figure 6A). Contrastingly, G4Dis treated and untreated cells showed no significant difference in protein levels. Reduction in NCL levels was confirmed through immunoblot. Using ImageJ to quantify the pixel density, we see approximately a 50% reduction in NCL protein for all treated samples (Figure 6B). Looking at the relative pixel densities and normalizing to their respective GAPDHs, G4WT-shNCL treated with Dox results in approximately 2-fold increase. In the G4Dis groups, there is no statistical difference between the treated and untreated.

NCL is predominantly found in the nucleolus, and by utilizing the shRNA, we looked at any difference in the distribution of mRNA containing G4 sites. HEK293L cells cotransfected with shNCL and the pA3F-G4WT/Dis clones, we fractionated the nuclear and cytoplasmic fractions, followed by the extraction of RNA from the respective fractions. Firstly, we confirmed that there was an expected downregulation of NCL through quantitative PCR (Figure 6C). The levels of G4WT and G4Dis mRNA were determined using sequence-specific primers (Figure 6D). In Dox-treated G4WT transfected cells, there was a greater proportion of mRNA exported to the cytoplasm and reduced nuclear retention compared to non-Dox treated cells. There was no statistically significant difference in G4Dis mRNA localization between Dox-treated and untreated cells. An increase in G4WT mRNA export to the cytoplasm under reduced NCL conditions may allow higher protein translation. Additionally, the altered localization of G4WT mRNA was confirmed through FISH (Figure 6G). The hybridization signal in the Dox-treated group was lower in the nucleolus. As expected, the nucleolus showed the highest levels of NCL, and interestingly, it appears that when NCL was downregulated, the hybridization signal increased in the cytoplasm. This may mean that when NCL was reduced, LANA G4 mRNA was exported into the cytoplasm. This corroborated the qPCR data presented in Figure 6D.

Using latently infected KSHV-positive BCBL-1 cells, we aimed to determine the effect of NCL on LANA levels in vitro. Transfecting shNCL in BCBL-1 cells and treating with Dox resulted in increased LANA levels (Figure 6E). Calculating the relative LANA pixel density, in the Dox-treated BCBL-1 cells, there is approximately a 2-fold increase in LANA protein levels (Figure 6F). This indicates that NCL plays a regulatory role in the expression of G4 sites containing LANA mRNA.

## 4. Discussion

KSHV establishes a life-long latent infection and can avoid detection from the host’s immune system. During the latent phase, KSHV limits its gene expression with abundant expression of LANA, which is essential for viral replication, distribution of viral genome to daughter cells, and avoiding the host’s immune detection [57]. LANA autoregulates its expression by regulating its promoter and inhibiting peptide synthesis and proteasomal degradation through two central region domains (CR2 and CR3) [10,58,59,60,61]. Here, we build on our previous findings and explore how NCL regulates the expression of LANA. We previously identified regions in LANA mRNA that form G4s. Additionally, we showed that hnRNP A1 acts as a helicase, disrupting the G4 secondary structure and resulting in an upregulation of LANA translation and antigen presentation [29].

G-quadruplexes have gained popularity as potential therapeutic targets for regulating viral genome replication and transcription [40,62,63]. G4s act as immunomodulatory structures in gamma herpesviruses. For example, in EBV and KSHV, G4s alter protein synthesis and antigen presentation and thus affect host immune detection [29,61]. Due to the homology of EBV’s EBNA1 being downregulated by NCL, we sought to see if KSHV’s LANA expression is regulated by NCL. Utilizing RNA oligonucleotides containing LANA’s mRNA region that forms a G4 structure, we showed that NCL interacts with LANA mRNA. To determine if this interaction occurred in cells, we transfected pA3F-G4WT and pA3F-G4Dis plasmids in HEK293L cells and performed RNA-CLIP to quantify the amounts of these mRNAs bound to NCL. Our data showed a specific association of LANA’s guanine-rich mRNA capable of forming a G-quadruplex to NCL. Basal but detectable binding of G4Dis mRNA with NCL could be due to some secondary structure in G4Dis mRNA that facilitated the interaction. Additionally, LANA mRNA in KSHV-infected cells, BCBL-1, showed a selective binding to NCL. Our LANA-Luciferase fusion protein confirmed that the NCL-LANA mRNA interaction decreases LANA translation. This is likely due to the ability of NCL to bind to the G-quadruplex in the mRNA and restrict them from being available for translation. Detection of protein levels through immunoblot confirmed a reduction in translation. Importantly, the localization of G4-containing mRNA through FISH confirmed that this interaction occurs within the nucleus. These results support that NCL binding to a G4 in LANA mRNA resulting in translational regulation through sequestration of mRNA in the nucleus.

**Figure 7:**
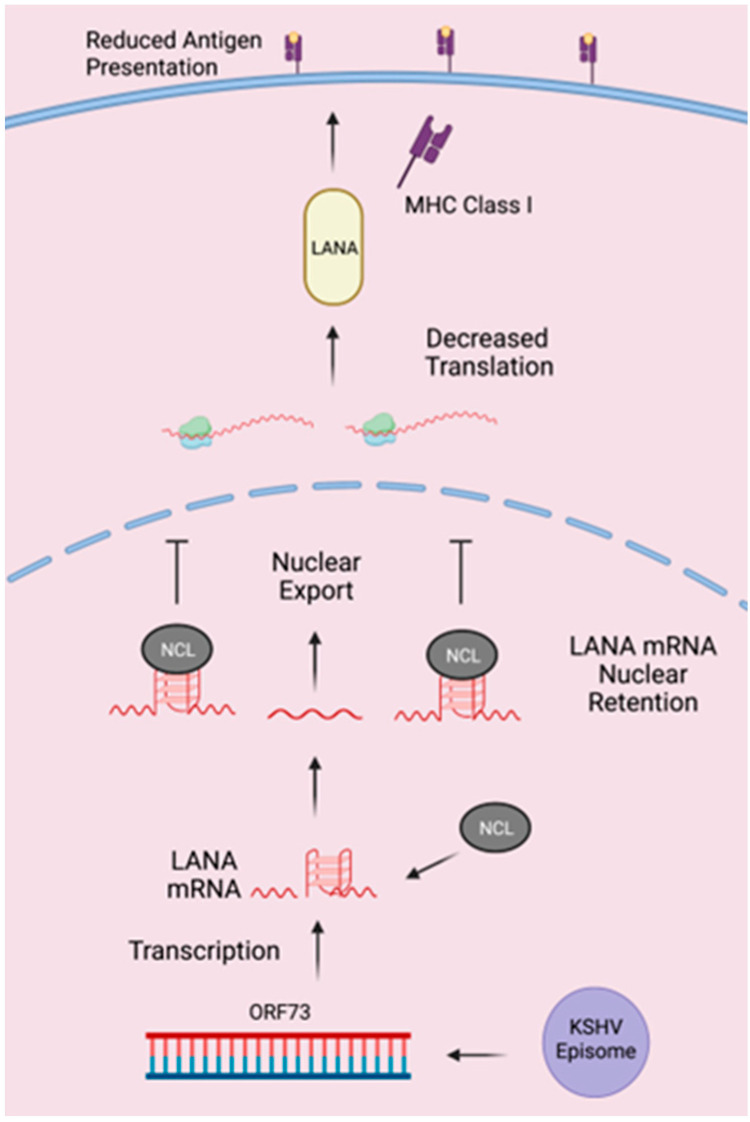
Proposed model of the regulation of LANA expression through NCL. NCL binds to the G-quadruplex of LANA mRNA, resulting in reduced levels of nuclear export of LANA mRNA into the cytoplasm. A lower transcript level for translation results in a reduced expression and, subsequently, lower antigen presentation on the cell surface.

LANA has been shown to regulate antigen presentation [10,13]. We previously found that stabilization/destabilization of the G-quadruplex in LANA regulates protein synthesis and antigen presentation. The nuclear LANA protein binds to the G-quadruplex in its mRNA, inhibiting the export of those bound mRNA molecules to the cytoplasm. This, in turn, reduces LANA protein translation, which is regulated by the levels of LANA in the cells. In cells with lower levels of LANA, hnRNP A1 binds to LANA mRNA, acts as a helicase, and disrupts the G-quadruplex within the mRNA. This leads to an export of LANA mRNA into the cytoplasm for its translation to maintain the viral genome. Cells having a threshold level of LANA in the nucleus, required for viral persistence, a relatively higher level of LANA than hnRNP A1 may become available to bind and retain the LANA mRNA in the nucleus [29]. This inhibits mRNA export into the cytoplasm, allowing the cycle to continue when LANA protein levels drop in the nucleus. In this paper, we showed that NCL can bind to LANA mRNA and localize in the nucleus, with a subset of NCL colocalizing with LANA mRNA. We further tested whether this interaction resulted in a reduced antigen presentation of these peptides to the cell surface in the presence of NCL. For this, cells expressing G4WT-OVA or G4Dis-OVA in the presence of NCL were utilized. Reduction in the presentation of G4WT-ova but not G4Dis-OVA in the presence of NCL confirmed the role of NCL in regulating expression. These results confirmed that stabilization of G-quadruplex in LANA mRNA can reduce expression and antigen presentation similar to what we reported previously [29]. Reduction in the expression of LANA is due to its mRNA being sequestered by NCL and reducing in LANA mRNA exported to the cytoplasm (Figure 7). Nucleolin is abundantly expressed in cells and is involved in rRNA maturation [32]. NCL binds to guanine-rich regions in coding and non-coding regions of various mRNAs [64]. Additionally, NCL binds to G-quadruplexes in both RNA and DNA, such as at the G4 in the LTR of HIV-1, to silence transcription [33]. Additionally, NCL binds to a G4 in the promotor of c-MYC, affecting its transcription [34,65]. As NCL has a myriad of roles in the cell, we studied the function of NCL in LANA expression by depleting its level by way of shRNA, which showed a reduction in the sequestration of LANA mRNA in the nucleus. This led to an increase in the expression levels of LANA. Sequestration of mRNA was determined by the fraction of cytoplasmic/nuclear fraction and the FISH assay confirming that NCL can regulate LANA mRNA translocation and expression.

The interaction of LANA mRNA with NCL in the nucleus may be attributed to the presence of nucleolar organizing regions (NORs), where NCL is notably abundant and actively participates in ribosomal biogenesis [66,67]. NCL, recognized for its RNA affinity and its significant role in organizing chromatin structure, could facilitate the localization of LANA RNA to these NORs. Furthermore, the nucleolus is integral to the interplay between KSHV and the host cell’s ribosomal biogenesis, a crucial aspect of the virus’ strategy to manipulate host cellular machinery for its replication. The synthesis and initial assembly of ribosomal RNA (rRNA) and ribosomal proteins into ribosomal subunits takes place in the nucleolus. KSHV actively modifies this ribosomal assembly process, leveraging it to support its replication needs [68,69]. During its lytic phase, KSHV disrupts normal ribosome biogenesis in the nucleolus, leading to the creation of specialized ribosomes, uniquely modified for efficient translation of viral mRNAs, especially those crucial for the virus’s late lytic cycle. This modification is hypothesized to result from KSHV-induced methylation patterns, tailoring ribosomes for viral protein synthesis. Thus, KSHV manipulates nucleolar activity, particularly rRNA modification, to produce ribosomes optimized for its replication, highlighting its sophisticated strategy to control host translation machinery [68].

Angiogenin, produced by KSHV infection, translocates to the nucleolus and increases the production of rRNA to enhance the survival of the infected endothelial cell [70]. LANA and angiogenin are shown to form a complex with the p53 protein, and is suspected that angiogenin and LANA hold p53 in the nucleus in an inactivated state [71,72]. Thereby, angiogenin could play a role in preserving the survival of cells infected with KSHV and in maintaining the virus in a dormant or latent state, potentially by influencing pathways that involve p53.

G-quadruplexes have become increasingly popular as therapeutic agents [40,62,63]. Regulating LANA expression through G-quadruplexes may offer a therapeutic target to control KSHV latency and removal of latent virus from the infected cells. The intricate cellular functions of NCL, as revealed in our findings, shed new light on the regulatory mechanisms governing LANA mRNA. Our prior demonstration of hnRNP A1’s helicase activity in unwinding a G4 structure in LANA mRNA has paved the way for a deeper exploration of the potential interplay between NCL and hnRNP A1 [29]. Our study not only signifies an important step in understanding the molecular dynamics, but also presents an important prospect—the existence of a regulatory axis involving NCL and hnRNP A1, with far-reaching implications for the life cycle of KSHV. Given the established functions of hnRNP A1 in mRNA metabolism and the novel insights provided by our findings, it is tempting to speculate on the existence of a regulatory axis involving NCL and hnRNP A1 that could impact the life cycle of KSHV. For example, NCL may influence the expression of hnRNP A1 through various post-transcriptional mechanisms or by affecting its protein stability. Alternatively, NCL could modulate the binding affinity of hnRNP A1 for LANA mRNA, thereby influencing the viral latency and reactivation process. Such interactions may not only be limited to the direct control of gene expression but could also encompass the broader epigenetic landscape, thus impacting viral replication and cell fate. This could offer insights into how viruses harness host cell machinery for their replication and survival, as well as reveal novel targets for antiviral therapy. The exploration of this regulatory interplay is, therefore, not only of basic scientific interest, but also of potential therapeutic relevance. Further studies employing a combination of biochemical, molecular, and systems biology approaches are warranted to dissect the layers of this complex interaction network. Through such multidimensional studies, we hope to illuminate the full scope of NCL’s and hnRNP A1’s roles in viral latency and reactivation, thereby enhancing our understanding of viral pathogenesis and informing the development of novel therapeutic strategies.

## Data Availability

The data presented here are available within this manuscript, Zareie and Verma, Viruses.

## Supplementary Materials

The following supporting information can be downloaded at: https://www.mdpi.com/article/10.3390/v15122438/s1, Table S1: LANA PQRSs.
