# Supplementary material for: Nucleolin Regulates the Expression of Kaposi’s Sarcoma-Associated Herpesvirus’ Latency-Associated Nuclear Antigen through G-Quadruplexes in the mRNA
Source: Viruses. 2023 Dec 15;15(12):2438. doi: 10.3390/v15122438 (PMC10747643; doi:10.3390/v15122438)
Supplement: Supplementary file 1 [file viruses-15-02438-s001.zip › viruses-2712197-supplementary.pdf]

LANA PQRSs with G-Scores

Position	Length	PQRSs	G-Score
26	19	GGTCGGGACGGAGCACCGG	17
915	18	GGCTGGCGAGGATAATGG	18
987	20	GGATGATGAGGAGGAGCAGG	15
1017	14	GGAGGACGAGGAGG	18
1038	17	GGAGGATGACGAGGAGG	15
1065	23	GGATGACGAGGAGGATGACGAGG	15
1110	17	GGAGGATGACGAGGAGG	15
1134	17	GGAGGATGACGAGGAGG	15
1158	20	GGAGGAGGACGAGGAGGAGG	21
1182	20	GGAGGAGGACGAGGAGGAGG	21
1227	29	GGACAATGAGGACGAGGAGGATGACGAGG	21
1260	29	GGACAAGAAGGAGGACGAGGAGGACGGGG	21
2091	29	GGATGAGCAGGAGCAGCAGGATGAGCAGG	21
2268	29	GGAGGAGCAGGAGCAGCAGGAGGAGCAGG	21
2316	23	GGAGCAGGAGTTAGAGGATCAGG	18
2352	23	GGAGCAGGAGCAGGAGTTAGAGG	18
2379	23	GGAGCAGGAGTTAGAGGAGCAGG	18
2427	23	GGAGTTAGAGGAGCAGGAGCAGG	18
2457	23	GGAGCAGGAGCAGGAGTTAGAGG	18
2484	23	GGAGCAGGAGTTAGAGGAGCAGG	18
2511	23	GGAGTTAGAGGAGCAGGAGGTGG	18
2547	23	GGAGGTGGAAGAGCAAGAGCAGG	9
2574	29	GGAAGAGCAGGAATTAGAGGAGGTGGAGG	21
2619	29	GGAGGAGCAGGAGGAGCAGGAGTTAGAGG	21
2652	29	GGAAGAGCAGGAAGAGCAGGAGTTAGAGG	21
2685	29	GGAAGAGCAGGAAGAGCAGGAGTTAGAGG	21
2718	29	GGAAGAGCAGGAGCAGCAGGAGTTAGAGG	21
2760	26	GGAGCAGCAGGGGGTGGAACAGCAGG	18
2822	30	GGTCGTCATCCGAGGACGAAATGGAAGTGG	14
3232	17	GGAGGAGTAAAGGCAGG	16
3445	20	GGGGAAAACCAAGGTCCTGG	13

Supplemental Table 1: LANA PQRSs
Table shows LANA PQRSs with corresponding G-Scores from QGRSMapper.
Overlapping PQRSs are excluded.
